# *PRKAG2* Variant, Motor Neuron Disease, and Parkinsonism: Fortuitous Association or a Potentially Underestimated Pathophysiological Mechanism?

**DOI:** 10.3390/muscles3030021

**Published:** 2024-07-25

**Authors:** Marco Orsini, Wladimir Bocca Vieira de Rezende Pinto, Paulo Sgobbi, Acary Souza Bulle Oliveira

**Affiliations:** 1 Faculdade de Medicina, Universidade Iguaçu (UNIG), Nova Iguapu 26275-580, RJ, Brazil; orsinimarco@hotmail.com; 2 Division of Neuromuscular Diseases, Federal University of São Paulo (UNIFESP), São Paulo 04021-001, SP, Brazil

**Keywords:** motor neuron disease, amyotrophic lateral sclerosis, parkinsonism, Wolff–Parkinson–White syndrome, cardiomyopathy, *PRKAG2*

## Abstract

A 72-year-old Brazilian woman presented with a 4-year history of rest tremors of the hands, followed by slowness of movement, and a diagnosis of idiopathic Parkinson’s disease. She was started on dopamine agonists with significant improvement. After three years, she complained about slowly progressive dysphagia, dysphonia, quadriparesis, and cramps and fasciculations. A neurological examination disclosed distal-dominant quadriparesis, dysarthria, atrophy and fasciculation of the tongue, global brisk tendon reflexes, fasciculations, bilateral ankle clonus, and moderate spasticity of the lower limbs. She had also palpitations, dyspnea, and one episode of paroxysmal atrial fibrillation. Electrocardiography revealed a short PR interval, a widened QRS complex, and the delta wave, suggestive of Wolff–Parkinson–White syndrome. Brain and spine MR imaging, a cerebrospinal fluid analysis, and general serum lab exams were unremarkable. Needle electromyography disclosed chronic denervation involving cervical, thoracic, lumbosacral, and bulbar levels associated with acute denervation, including positive sharp waves, fasciculations, and fibrillation potentials. This patient fulfilled the diagnostic criteria for amyotrophic lateral sclerosis associated with parkinsonism. A broad next-generation sequencing-based panel disclosed the presence of the novel heterozygous variant c.1247C > T (p.Pro416Leu) in the *PRKAG2* gene (NM_016203.4). Clinicians must be aware of the possibility of *PRKAG2* variants in complex clinical scenarios associating cardiac arrhythmia, preexcitation syndromes, hypertrophic cardiomyopathy, motor neuron disease, and parkinsonism.

## 1. Introduction

Complex intracellular pathways have been associated with the pathophysiology of different neurodegenerative disorders, such as frontotemporal dementia, motor neuron disease (MND)/amyotrophic lateral sclerosis (ALS), atypical parkinsonism, and inclusion body myopathy [1,2]. The 5′adenosine monophosphate (AMP)-activated protein kinase (AMPK) is activated by different intracellular catabolic events associated with ATP level decrease and AMP increase to inhibit ATP-depleting reactions and mechanisms. One important structure of the AMPK complex is the noncatalytic subunit gamma (such as the PRKAG2 protein), as well as a catalytic alpha subunit and a noncatalytic beta subunit. AMPK is also associated with the regulation of autophagic processes, which are directly correlated with age-associated neurodegenerative disorders and involved in the modulation of metabolic processes, such as glycolysis and glucose uptake [3,4].

*PRKAG2* (7q36.1) pathogenic variants have been previously linked to PRKAG2 cardiac syndrome or “AMPK disease”, including Wolff–Parkinson–White syndrome (WPWS), lethal congenital glycogen storage disease of the heart, and hypertrophic cardiomyopathy type 6 [4,5,6,7]. Nonetheless, there are cardiovascular and systemic phenotypes that are still probably under-recognized in clinical practice. In other contexts, single-nucleotide polymorphisms in the *PRKAG2* gene have been identified to confer susceptibility or as potential risk factors for different clinical conditions, such as atopic dermatitis [8], diabetes in the elderly, and cognitive decline [9]. *PRKAG2* variants have also been observed in association with IgA nephropathy [10] and liver cirrhosis [11]. The association of pathogenic variants leading to specific neurodegenerative phenotypes has not been previously described in the literature, despite the well-known role of AMPK and other kinases in the pathophysiology of several neurological diseases, including neurodegenerative diseases, such as MND/ALS and Alzheimer’s disease [12]. It should indeed be the subject of discussion based on future scientific evidence whether variants correlated with the *PRKAG2* gene in relation to possible neurological complications will have a more relevant role as risk factors or as mechanisms correlated with causality. We describe, herein, the case of a previously healthy elderly woman who presented with a new and complex late-onset phenotype with the association of WPWS, MND/ALS, and parkinsonism.

## 2. Case Presentation

A 72-year-old Brazilian woman presented with a 4-year history of rest tremors of the hands, followed by slowness of movement, who was evaluated by another neurological center and diagnosed with idiopathic Parkinson’s disease. She was started, at that time, on dopamine agonists with significant improvement in tremor and movement amplitude and speed. However, after three years, she complained about slowly progressive dysphagia, dysphonia, quadriparesis, and cramps and fasciculations. She had no known significant previous chronic disorders. No relevant history of cardiovascular, pulmonary, renal, orthopedic, or neurological diseases was known. Her family history was also unremarkable regarding neurological, neuromuscular, and cardiovascular disorders. A neurological examination disclosed distal-dominant quadriparesis, dysarthria, atrophy and fasciculation of the tongue, global brisk tendon reflexes, diffuse fasciculations, bilateral ankle clonus, and moderate spasticity of the lower limbs. No signs of cognitive decline were detected. Thus, a neurological examination showed typical findings seen in the context of upper and lower motor neuron involvement, in addition to the previous onset of parkinsonism.

At the same time as the neurological evaluation and diagnostic workup, this patient also had symptoms of palpitations, dyspnea, and one episode of tachycardia with paroxysmal atrial fibrillation, which underwent catheter ablation after the first clinical evaluation by the cardiologist. During that time, the patient performed an extensive diagnostic workup, including electrocardiography studies, which revealed a short PR interval, a widened QRS complex, and the characteristic delta wave, which are typically seen in preexcitation syndromes such as Wolff–Parkinson–White syndrome (WPWS).

Neuroimaging studies of the brain and spinal cord (magnetic resonance), cerebrospinal fluid analysis, and general serum lab exams were unremarkable. A muscle biopsy was not performed during the diagnostic work-up. Nerve conduction studies were also unremarkable. Needle electromyography disclosed the presence of chronic denervation involving the cervical, thoracic, lumbosacral, and bulbar levels, which is associated with acute signs of denervation, including positive sharp waves, fasciculations, and fibrillation potentials. Thus, this patient fulfilled modified El Escorial, Gold Coast, and Awaji-shima diagnostic criteria for amyotrophic lateral sclerosis (ALS) associated with parkinsonism. This complex presentation was, at first, considered a sporadic presentation of MND, as there were no other familial cases of ALS established in the family.

Due to the rare association of complex cardiological and neurodegenerative phenotypes seen, especially in a late-onset and sporadic presentation, broad genetic testing to evaluate a common pathogenetic mechanism for this complex phenotype was requested. A broad next-generation sequencing (NGS)-based multigene panel, including genes associated with MND/ALS, parkinsonism, cardiac arrhythmias, and cardiomyopathy, was performed. After the extraction of genomic DNA, the DNA library was prepared using a target enrichment method and capture of target regions (coding DNA and splicing sites), and then sequencing was carried out on an Illumina platform. Genetic testing disclosed the presence of the novel heterozygous variant c.1247C > T (p.Pro416Leu) in the *PRKAG2* gene (NM_016203.4), fulfilling PM2, PP3, and PP4 American College of Medical Genetics and Genomics (ACMG) criteria [13]. Different “in silico” prediction tools indicate a deleterious effect of this variant, including SIFT, MutationTaster2, Revel, AlphaMissense, Varity, MUT Assessor, DANN, MetaLR, Primate AI, BayesDel, GenoCanyon, and fitCons. This very rare variant is absent from the ClinVar database and was not found in any of the different populational genomic databases, such as in gnomAD. *C9orf72* gene hexanucleotide repeat expansion was not evaluated during the diagnostic workup. We, therefore, considered, in this context, the *PRKAG2* variant directly related to the cardiac phenotype described and possibly associated with the complex neurological picture.

## 3. Discussion

Different cardiac clinical conditions associated with the *PRKAG2* gene and their pathophysiological mechanisms have been recognized (Table 1). The pathogenic variants of the *PRKAG2* gene identified are mainly related to missense variants and inframe indels, mainly related to the WPWS phenotype alone or associated with hypertrophic cardiomyopathy and the phenotype of autosomal-dominant familial hypertrophic cardiomyopathy type 6 secondary to nonlysosomal glycogenosis during late adolescence or early adulthood. Variants generally relate to rare clinical contexts associated with complete penetrance and autosomal dominant inheritance, although sporadic cases are recognized [5,6,7]. Only in rare clinical contexts of *PRKAG2*-associated syndromes do extracardiac manifestations such as progressive structural myopathy occur in the context of systemic glycogen storage disease, including forms associated with the formation of polyglucosan body storage [5,14]. Most variants are located in the CBS (Bateman) domain and have been associated with distinct cardiac involvement; however, rare variants in the non-CBS domain have also been reported [5]. From studies in a transgenic mouse model (later correlated with different evidence from individuals with *PRKAG2* variants), it was possible to observe that changes in AMPK activities were related to greater accumulations of cardiac glycogen and higher degrees of left ventricular hypertrophy, as well as atrioventricular conduction via alternative pathways with ventricular preexcitation and sinus node dysfunction due to annulus fibrosis infiltration by cytosolic vacuoles rich in glycogen-filled enlarged cardiomyocytes [15,16,17]. In cases associated with polyglucosan storage myopathies, typical polyglucosan aggregates may form with an amylopectin-like polysaccharide structure with a periodic acid-Schiff-positive and diastase-resistant profile, which can aggregate as polyglucosan bodies [18].

The threefold increase in the *PRKAG2* gene expression with raised AMPK levels is related to an increase in beta-amyloid accumulation in the brains of patients with Alzheimer’s disease [19]. Thus, the higher the expression of the *PRKAG2* gene, the more important the level of AMPK that occurs, and then, consequently, the abnormal metabolism and processing of the precursor protein occurs, leading to increased formation of beta-amyloid in individuals with Alzheimer’s disease. There has also been a previous association of *PRKAG2* polymorphisms with cognitive decline in the elderly [9]. No previous description related to motor neuron disease or parkinsonism has been established in the literature. Furthermore, different kinases (sharing characteristics similar to AMPK) have already been associated with mechanisms of dysfunction related to ALS, regulating axonal transport by interacting with other proteins like neurofilaments, microtubules, and anterograde and retrograde transport, participating in the ubiquitin–proteasome systems and autophagy pathways and the regulation of critical neuroinflammatory mediators [12]. Some of these main kinases related to ALS include TANK-binding kinase (coded by *TBK1* gene), NIMA-related kinase 1 (coded by the *NEK1* gene), and Erb-B2 receptor tyrosine kinase 4 (coded by the *ERBB4* gene) [12]. Some of these kinases are implicated in specific roles and interactions, such as retrograde transport by dynein, anterograde transport by kinesin, and adapters, neurofilaments, and microtubules [12]. It has also been previously shown that human motor neurons with transactive response DNA-binding protein 43 (TDP-43) pathological mislocalization have increased AMPK activation; in addition, *SOD1*-ALS mouse models have revealed the upregulation of the AMPK-mediated phosphorylation of compounds linked to autophagy in the spinal cord [12,20]. Furthermore, in TDP-43 transgenic mouse models, AMPK activity inhibition gave rise to the slowed and delayed progression of the disease [12,20]. In another TDP-43 transgenic mouse model, both in presymptomatic and symptomatic animals, there was severely reduced AMPK activity in the motor neurons of the spinal cord and the brain cortex [12,21]. Previous studies have also demonstrated, through protein interaction network analysis, the pathophysiological correlation of interaction with the sequestosome 1 (SQSTM1) protein [22], which is well-correlated with the pathophysiology of different systemic degenerative and neurodegenerative diseases involving multisystemic proteinopathies, such as frontotemporal dementia, MND/ALS, Paget disease of the bone, and myopathy [1,2].

Although *PRKAG2* gene expression occurs in the central nervous system in different topographies, there are no data available regarding the existence of glycogen or polyglucosan body deposits seen at autopsy in patients with *PRKAG2* pathogenic variants. There is, however, current evidence demonstrating the expression of the *PRKAG2* gene (ENSG00000106617.13) in different locations of the central nervous system, most significantly in the basal ganglia in the nucleus accumbens, putamen, and caudate, although it also occurs in other regions (the Human Protein Atlas) [23]. Likewise, the AMPK protein is currently implicated in the pathophysiology of different processes related to neurological diseases, especially in pathways related to mammalian target of rapamycin (mTOR) and in the regulation of autophagy mechanisms, modulating short-term metabolism cellular enzymes in the cholesterol and fatty acid biosynthesis pathways, and pathways related to mitochondrial biogenesis through the peroxisome proliferator-activated receptor (PPAR) and PPAR coactivator-1 alpha (PGC1alpha) [24]. In a similar clinical context, in adult polyglucosan body disease (APBD), due to variants in the *GBE1* gene, neuropathy, dysautonomia, parkinsonism, cognitive decline, and leukoencephalopathy can be observed in varying degrees in affected adult individuals [25]. Compound heterozygous or homozygous variants in the *GBE1* gene have been also associated with myopathic, hepatic, and cardiomyopathic involvement seen in glycogen storage disease type IV (Andersen disease) [25].

Genetic variants in several genes related to inherited metabolic diseases, such as Gaucher disease, and heterozygous polymorphisms, such as in the *GBA* gene, have been previously identified as risk factors for the occurrence of parkinsonism and even MND. In these contexts, these variants usually represent additional risk factors and not specific monogenic pathophysiological mechanisms that occur along with the clinical neurological presentation. The contribution role of variants in the *GBA* gene within the neurodegenerative process due to endolysosomal dysfunction in MND/ALS has been established [26,27]. In parkinsonism, there are both stress responses in the endoplasmic reticulum with unfolded protein response and lysosomal dysfunction with the partial loss of glucocerebrosidase activity causing the accumulation of complex glycosphingolipids, glycosylceramide, and glycosylsphingosine [28,29]. There are, however, other forms of MND with or without associated parkinsonism that have a specific monogenic basis related to genes linked to inherited neurometabolic diseases, such as GM2 gangliosidosis and APBD [30].

In view of the abovementioned information, it may be questioned whether the new variant identified in *PRKAG2* is associated only with the context of cardiac arrhythmia without correlation with the neurological picture, or whether the new variant could be related to as a risk factor or a causal etiopathogenic mechanism associated with neurodegeneration. Despite previous studies indicating the possibility of *PRKAG2* as a candidate gene for early Parkinson’s disease [31], more robust evidence is still necessary, and more data regarding the role of AMPK in the pathophysiology of MND/ALS and Parkinson’s disease will enable a better understanding of pathogenesis [24]. Clinicians must be aware of the possibility of *PRKAG2* variants in complex clinical scenarios associated with cardiac arrhythmia, preexcitation syndromes, hypertrophic cardiomyopathy, MND/ALS, and parkinsonism. Future evidence resulting from observations in new genetic epidemiological studies in populations with sporadic and familial ALS or parkinsonism (i.e., genome-wide association studies), are still needed. The concomitant occurrence of neurological symptoms and signs in patients with Wolff–Parkinson–White syndrome, hypertrophy cardiomyopathy, or atrioventricular blocks should be carefully evaluated by clinicians in the near future during diagnostic work-up. Data obtained from autopsy studies in the future in the population with cardiac involvement may also contribute to evaluating the topography of the basal ganglia, the anterior horn of the spinal cord, and the primary motor cortex, seeking to characterize the presence of intraneuronal glycogen accumulation and polyglucosan bodies.

This case report certainly presents the practical limitations of reporting the presence of a genetic variant in the context of a late-onset cardiac phenotype that is highly suggestive of being correlated with this variant; however, it is within a scenario of complex neurological manifestations, including MND/ALS and parkinsonism. The presence of the highlighted variant certainly does not exclude the possibility of other pathophysiological mechanisms in the origin of the neurological symptoms described. However, the existence of similar pathophysiological mechanisms observed in other inherited metabolic diseases, which can potentially be seen in cases similar to the one described, should highlight the importance of considering the described variant in potential causal association. It must be emphasized that future confirmations in new scenarios are necessary, including genome-wide association studies, other population-based genetic studies, and new case reports or case series. Further studies are still necessary to evaluate the role of AMPK in some possibly under-represented neurological conditions, such as MND/ALS.

## 4. Conclusions

*PRKAG2*-related syndromes are associated with complex cardiac arrhythmia, WPWS, hypertrophic cardiomyopathy, and rarely, polyglucosan storage myopathy. Despite the well-established role of AMPK in the pathophysiology of several central nervous system diseases, a direct monogenic basis has not been previously defined. Our case report describes a sporadic late-onset presentation of WPWS associated with parkinsonism and ALS in the context of *PRKAG2* variant, thus highlighting the potential importance of AMPK and *PRKAG2* variant in the pathophysiology of late-onset MND/ALS and parkinsonism.

## Figures and Tables

**Table 1:** Summary of the main clinical aspects associated with *PRKAG2*-related disorders [4,5,6,7].

Main Phenotypes Associated with *PRKAG2* Variants	Pattern of Inheritance	Age at Onset	Clinical Presentation
Familial hypertrophic cardiomyopathy type 6 (MIM #600858)	Autosomal dominant	Variable; generally juvenile or adult onset	Hypertrophic cardiomyopathy; atrial fibrillation, ventricular preexcitation syndrome; atrioventricular block; skeletal myopathy (rare)
Wolff–Parkinson–White syndrome (MIM #194200)	Autosomal dominant	Variable; childhood or early adulthood onset	Ventricular preexcitation syndrome; typical delta wave; short PR interval; widened QRS complex; supraventricular tachycardia, atrial fibrillation; palpitation; sudden cardiac death
Lethal congenital glycogen storage disease of the heart (MIM #261740)	Autosomal dominant	Neonatal onset	Hypertrophic cardiomyopathy; vacuolar cardiomyopathy, congestive heart failure; association with preexcitation syndrome/Wolff–Parkinson–White syndrome; skeletal myopathy; severe presentation

## Data Availability

The data which supports the findings showed in this study have been presented in the article.

## Abbreviations

ACMG	American College of Medical Genetics and Genomics
ALS	Amyotrophic lateral sclerosis
AMP	5′adenosine monophosphate
AMPK	AMP-activated protein kinase
APBD	Adult polyglucosan body disease
MND	Motor neuron disease
MRI	Magnetic resonance imaging
mTOR	Mammalian target of rapamycin
NGS	Next-generation sequencing
PGC1alpha	PPAR coactivator-1 alpha
PPAR	Peroxisome proliferator-activated receptor
PRKAG2	Protein kinase, AMP-activated, noncatalytic, subunit gamma 2
SQSTM1	Sequestosome 1
TDP-43	Transactive response DNA-binding protein 43
WES	Whole-exome sequencing
WPWS	Wolff–Parkinson–White syndrome
